# Differences in globus pallidus neuronal firing rates and patterns relate to different disease biology in children with dystonia

**DOI:** 10.1136/jnnp-2015-311803

**Published:** 2016-02-04

**Authors:** V M McClelland, A Valentin, H G Rey, D E Lumsden, M C Elze, R Selway, G Alarcon, J-P Lin

**Affiliations:** 1 Department of Clinical Neurophysiology, King's College Hospital NHS Foundation Trust, London, UK; 2 Department of Basic and Clinical Neuroscience, King's College London, London, UK; 3 Department of Human Physiology, Faculty of Medicine, Complutense University, Madrid, Spain; 4 Centre for Systems Neuroscience, University of Leicester, Leicester, UK; 5 Rayne Institute, King's College London, London, UK; 6 Complex Motor Disorder Service, Evelina Children's Hospital, Guy's and St Thomas’ NHS Foundation Trust, London, UK; 7 Department of Statistics, University of Warwick, Coventry, UK; 8 Department of Functional Neurosurgery, King's College Hospital NHS Foundation Trust, London, UK

## Abstract

**Background:**

The pathophysiology underlying different types of dystonia is not yet understood. We report microelectrode data from the globus pallidus interna (GPi) and globus pallidus externa (GPe) in children undergoing deep brain stimulation (DBS) for dystonia and investigate whether GPi and GPe firing rates differ between dystonia types.

**Methods:**

Single pass microelectrode data were obtained to guide electrode position in 44 children (3.3–18.1 years, median 10.7) with the following dystonia types: 14 primary, 22 secondary Static and 8 progressive secondary to neuronal brain iron accumulation (NBIA). Preoperative stereotactic MRI determined coordinates for the GPi target. Digitised spike trains were analysed offline, blind to clinical data. Electrode placement was confirmed by a postoperative stereotactic CT scan.

**Findings:**

We identified 263 GPi and 87 GPe cells. Both GPi and GPe firing frequencies differed significantly with dystonia aetiology. The median GPi firing frequency was higher in the primary group than in the secondary static group (13.5 Hz vs 9.6 Hz; p=0.002) and higher in the NBIA group than in either the primary (25 Hz vs 13.5 Hz; p=0.006) or the secondary static group (25 Hz vs 9.6 Hz; p=0.00004). The median GPe firing frequency was higher in the NBIA group than in the secondary static group (15.9 Hz vs 7 Hz; p=0.013). The NBIA group also showed a higher proportion of regularly firing GPi cells compared with the other groups (p<0.001). A higher proportion of regular GPi cells was also seen in patients with fixed/tonic dystonia compared with a phasic/dynamic dystonia phenotype (p<0.001). The GPi firing frequency showed a positive correlation with 1-year outcome from DBS measured by improvement in the Burke-Fahn-Marsden Dystonia Rating Scale (BFMDRS-m) score (p=0.030). This association was stronger for the non-progressive patients (p=0.006).

**Interpretation:**

Pallidal firing rates and patterns differ significantly with dystonia aetiology and phenotype. Identification of specific firing patterns may help determine targets and patient-specific protocols for neuromodulation therapy.

**Funding:**

National Institute of Health Research, Guy's and St. Thomas’ Charity, Dystonia Society UK, Action Medical Research, German National Academic Foundation.

## Introduction

Dystonia is “a movement disorder characterised by sustained or intermittent muscle contractions causing abnormal, often repetitive, movements, postures or both”.^1^ ^2^ In primary dystonia, the dystonic movements are the only feature of neurological disease and structural neuroimaging is normal. Secondary dystonias arise from a disease process or insult to the brain and can be further divided into those with static lesions (eg, following hypoxic ischaemic encephalopathy (HIE), extreme prematurity, infection, a metabolic disturbance or vascular event) and those with progressive disorders. The latter group includes the heredodegenerative dystonias, which are characterised by neurodegeneration, for example, pantothenate kinase 2 (PANK2) Deficiency, the most common neurodegeneration with brain iron accumulation (NBIA) disorder.^3^ ^4^ In childhood, dystonia is most often secondary rather than primary,^5^ the movement disorder is often very severe and the proportion of life lived with dystonia (PLD) is high.^6–9^ Many children never have a period of normal motor development^7^ and conventional medical management is often unsuccessful or poorly tolerated.^9^ ^10^ Dystonia in childhood remains an area of unmet need in disordered developmental motor control, the exploration of which is central to our understanding of emergent human basal ganglia function and the associated distributed networks.

Deep brain stimulation (DBS) of the globus pallidus interna (GPi) is a well-established management for medically refractory dystonia in adults and children.^11–14^ Patients with primary dystonia may show up to 88% improvement in the movement scale of the Burke-Fahn-Marsden Dystonia Rating Scale (BFMDRS-m). Patients with secondary dystonia show greater variability in response, ranging from 0% to 71% improvement, although the mean response is usually less than 25%.^7^ ^11^ ^12^ ^14–21^ In addition to clear clinical benefits, DBS affords the opportunity to record neuronal activity directly from the human basal ganglia, providing key insights into the underlying pathophysiology of dystonia. Microelectrode recordings have demonstrated reduced mean discharge rates from the GPi in adults with dystonia compared with the non-human primate or humans with Parkinson's disease.^22–25^ However, no consistent differences in GPi firing have been demonstrated between primary versus secondary dystonia,^26^ ^27^ which is surprising, given the differential response to DBS. However, previous comparisons have only involved small numbers of patients with secondary dystonia, and may have been underpowered to detect a difference. We analysed microelectrode recordings from the GPi and globus pallidus externa (GPe) in 44 children undergoing DBS for severe generalised dystonia, aiming to test the hypothesis that pallidal firing differs between different dystonia aetiologies. We found clear differences between aetiological groups, demonstrating that pallidal firing patterns correlate with disease biology. This has clear clinical relevance to the rapidly expanding field of neuromodulation, where understanding the disease-specific pathophysiology is essential for the development of more individualised neuromodulation approaches.

## Materials and methods

### Patients

This was a retrospective analysis of recordings and assessments performed as part of standard clinical practice, and thus formal ethical approval was not required under National Health Service (NHS) research governance arrangements. All families gave written consent for the surgical procedures.

The 44 patients were examined by a consultant paediatric neurologist with expertise in movement disorders (J-PL) and classified as primary (n=14) (ie, dystonic movements were the only feature of the neurological disease and structural neuroimaging was normal); secondary static (n=22), ie, the movement disorder was symptomatic of a known (non-progressive) brain insult, and secondary progressive dystonia due to NBIA (n=8) diagnosed by clinical features, classical imaging findings of iron deposition in the globus pallidus and nigrostriatal tract and confirmation of genetic mutation in PANK2. These groups were chosen to allow comparison with previously published literature from our own group and others. Each patient was also classified according to the recently proposed consensus classification for dystonia^1^ as shown in online supplementary table S1.

Dystonia severity was assessed using BFMDRS-m at baseline. DBS outcome was expressed both as percentage and absolute improvement in BFMDRS-m from baseline to 1 year postoperatively. The assessments were made using video-taped evaluations, reviewed by two clinicians (not blind to patient condition) and a score agreed. PLD was calculated as the duration of dystonia divided by age at the time of DBS.

### Surgery and electrophysiological recording

Surgery was performed under isoflurane general anaesthesia. Prior to microelectrode recordings, isoflurane was reduced to a standard level of 0.6–0.8 minimum alveolar concentration in all patients to provide conditions of ‘light anaesthesia’. Stereotactic MRI was performed preoperatively under anaesthesia with a Leksell G Frame in place to determine coordinates targeted in the posterolateroventral GPi. Prior to insertion of the stimulating electrode, microelectrode data were obtained to guide electrode tip position.^28^ This was usually single pass, but if no active cells were identified, a fresh electrode was passed along the same trajectory to rule out an equipment fault or false passage down a lamina. Single cell and multiunit neuronal recordings were made at −10, −7.5, −5, −4, −3, −2, −1, 0, +1, and +2 mm from the MRI-determined GPi target, analogue bandpass filtered between 500 and 5000 Hz and digitised at 24 KHz with a four-channel Leadpoint system (Medtronic, Minneapolis, Minnesota, USA). The presence or absence of active cells at each level assisted in identification of the GPe, medial medullary lamina and GPi, thus guiding the electrode tip position. Specifically, we intraoperatively used a combination of the depth of microelectrode advancement (mm above or beyond the planned target in the posterolateroventral GPi) and the identification of a period of microelectrode silence between GPe and Gpi (taken as representing the medial medullary lamina) to distinguish between GPe and GPi. Once the target had been confirmed or amended, the microelectrode was withdrawn and the stimulating electrode inserted along the same track (quadripolar DBS electrode model 3389, Medtronic). Final electrode placement was confirmed by a postoperative stereotactic CT scan, under the same general anaesthetic, fused with the intraoperative in-frame presurgical MRI. The pulse generator was then inserted (Soletra, Kinetra, Activa PC or Activa RC pulse generators, Medtronic).

### Offline data analysis

All analyses were performed blind to the patients’ clinical details. The location of each recording level (GPi, medial medullary lamina, GPe, Putamen) was determined from a combination of the target position and intraoperative neuronal recordings, as outlined above, cross-referenced with the actual electrode depth on the postoperative, in-frame CT scan. The possibility of inaccurate designation of some cells cannot be entirely excluded. However, cells were excluded from the analysis if their localisation was uncertain to reduce the number of inaccurately designated cells to a minimum. The digitised neural recordings were imported into MATLAB for offline analysis using *Wave_clus*, a spike-sorting algorithm that uses the wavelet transform for feature extraction from the spike waveforms and superparamagnetic clustering to discriminate the spikes from different units.^29^ Only extracellular action potentials with stable waveforms and a good signal to noise ratio were selected for processing. The threshold for spike detection was set at four times the estimated SD of the noise level.

**Figure 1:**
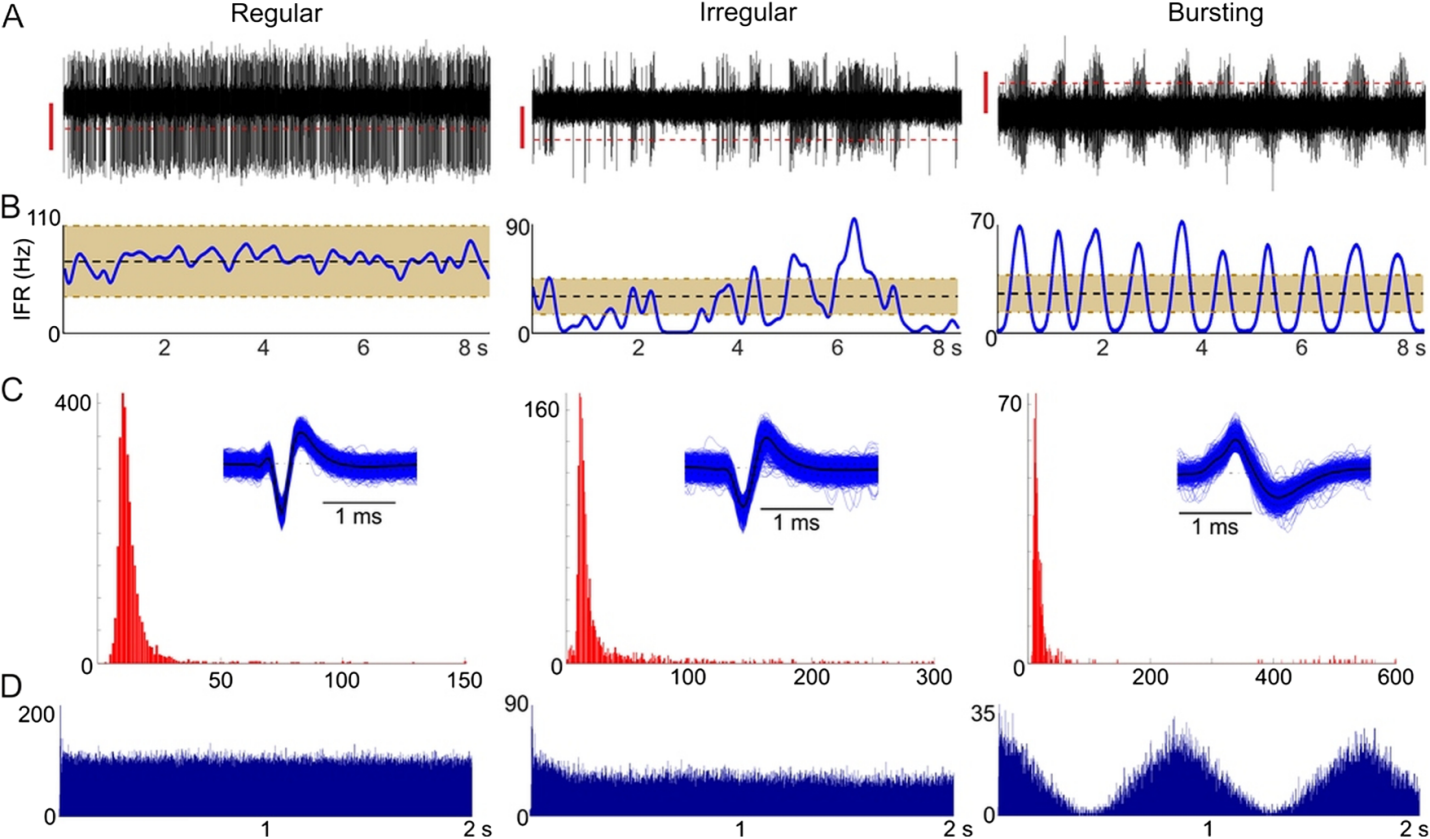
Analysis of neuronal properties. Examples of regular, irregular and bursting cells: (A) raw activity (500–5000 Hz) showing the threshold for spike detection (dashed line). The vertical red lines represent 20 μV. (B) Instantaneous firing rate (IFR) over the same time period. Shaded area illustrates the mean firing rate (μ)±0.5 μ. (C) Interspike interval (ISI) distribution (note the different scales in the x-axes reflecting the greater dispersion of ISIs for irregular and bursting cells). Inset shows the superimposed waveforms of the detected spikes. (D) Autocorrelation histogram showing the typical flat profile of a regular cell, rhythmic peaks in a rhythmic bursting cell and an intermediate profile for an irregular cell.

The following neuronal properties were analysed: mean firing rate (μ), interspike interval (ISI) distribution and instantaneous firing rate (IFR). The IFR was obtained by computing the convolution between the spike train and a Gaussian kernel sampled at 24 kHz with a total width of 500 ms (truncated at 1% amplitude). In addition, the co-efficient of variation of the ISI (ISI-CV) was calculated as the SD of the ISI divided by the mean ISI, similar to the measure of dispersion used by Vitek and colleagues.^23^ Cells were classified as regular, irregular or bursting through a combination of visual inspection of the firing pattern and the ISI distribution,^25^ and analysis of the IFR (figure 1). The degree of regularity was defined as the percentage of IFR samples falling within μ±0.5 μ for that cell. A regular cell was defined as one in which the IFR fell within μ±0.5 μ on more than 70% of the samples (figure 1A). Cells not meeting this criterion were classed as non-regular (figure 1B). We performed an additional assessment of regularity using the ISI-CV, whereby cells with an ISI-CV of one or less were classified as regular and cells with an ISI-CV of greater than one were classified as non-regular.

Bursts can be defined as a short period containing a high number of discharges separated by intervals of reduced or no activity. We defined bursting cells as those in which the bursts were rhythmic and of low frequency (<10 Hz), as per Magarinos-Ascone *et al*.^25^ Bursts can also occur at random in irregular cells (figure 1B). However, for bursting cells, the IFR shows a characteristic rhythmic fluctuation between relatively high frequencies and zero (figure 1C). Only those cells showing a rhythmic fluctuation of their firing rate were classified as bursting cells in this analysis (figure 1C).

### Statistical analysis

Prior to data analysis, our four key hypotheses were that GPi and/or GPe cell firing frequencies and/or firing patterns would differ between different types of dystonia. We use the Holm–Bonferroni method to keep the family-wise error rate for these four key hypotheses below 0.05 and give multiple testing-corrected p values in the Results section.^30^ During data analysis, secondary hypotheses were developed that firing rates or patterns might differ with dystonia phenotype and that severity of the motor disorder and/or outcome from DBS might be associated with GPi or GPe firing rates.

**Figure 2:**
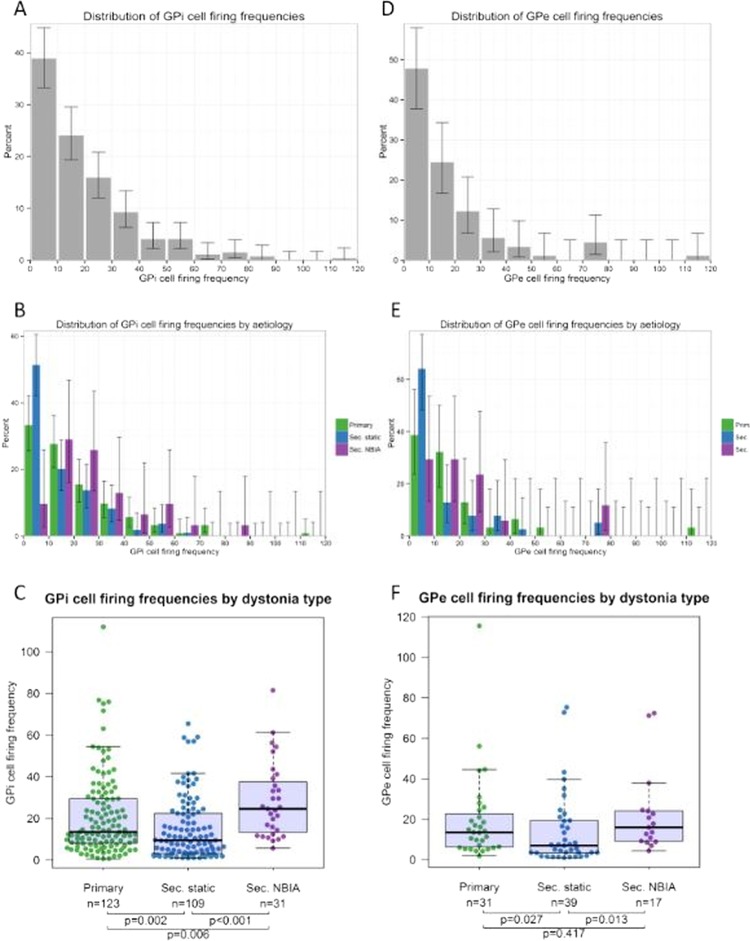
Distribution of GPi (A–C) and GPe (D–F) cell firing frequencies. (A) Histogram shows percentage of GPi cells within each 10 Hz frequency bin. Lines show the 95% Agresti-Coull add-4 CIs. (B) Distribution of GPi cells by firing frequency for each of the three main subgroups. (C) Box-and-whisker plot of GPi cell firing frequencies for each dystonia group. Circles show firing frequency of individual cells. Horizontal lines within boxes show the group median, boxes show the IQR, and whiskers show the full range. (D–F) Equivalent figures for GPe cells. The Kruskal-Wallis test showed a significant difference in the median firing frequency across the three groups for both GPi cells (p=0.00003) and GPe cells (p=0.03). The p values show the results of Mann-Whitney tests between groups. GPe, globus pallidus externa; GPi, globus pallidus interna.

Statistical analyses were performed in SPSS (IBM SPSS Statistics 21) and the R language and environment for statistical computing (V.3.01, R Development Core Team).^31^ The data were non-normally distributed. Relationships between numerical variables were tested using the Spearman correlation. Kruskal-Wallis and Mann-Whitney tests were used to investigate differences in numeric data between groups. For histograms, Agresti-Coull add-4 CIs were provided to compensate for smaller sample sizes in some groups.^32^ For the association between categorical variables, the χ^2^ test was used. Where outliers were identified, sensitivity analyses were performed to check that these had no undue influence on the results. We confirmed our findings for figure 2C, F and online supplementary figure S1 using mixed effects models (see online supplementary materials).

## Results

Clinical data are summarised in online supplementary table S1. Median age was 10.7 years (range 3.3–18.1). Age did not differ across groups (Kruskal-Wallis test p=0.162). Accuracy of electrode placement in our cohort is reported elsewhere and compares favourably with other groups.^28^ We identified 263 GPi and 87 GPe cells from 81 electrode trajectories. In three patients, no active cells were identified (two primary and one secondary static). Since a technical problem cannot be excluded in these cases, these patients were excluded from further analysis. However, the possibility of cells with no spontaneous activity (or ‘silent cells’) is also considered. In one patient with NBIA, four trajectories were made, as the first pass yielded no cells on either side, but cells were identified on the second pass on each side. Thus, there were eight ‘silent penetrations’ in total, spread across all main subgroups. In two patients, no active cells were identified in the GPi but clear cells were identified in the GPe; in 10 patients, no active cells were identified in the GPe but clear cells were found in the GPi. The identification of active cells in a part of these trajectories made a technical explanation less likely and these patients were therefore included in the analysis. The possibility of ‘silent’ GPi or GPe cells (respectively) with no spontaneous activity is a stronger consideration in these cases. However, it is important to note that the aim of our recordings was to identify the inferior margin of the GPi, and therefore the path through the GPe was not strongly considered in the targeting of the trajectory. Some paths may have had very little GPe involved.

Table 1 shows the number and firing frequency of GPi and GPe cells identified for each subgroup. The mean number of GPi cells per penetration was 3.4 and the mean number of GPe cells per penetration was 1.3. The number of GPi cells per trajectory did not differ across the three main subgroups, although there was a trend towards a higher number of cells per trajectory in the primary group (Kruskal-Wallis test p=0.070). Each trajectory was divided into recordings taken at the different levels approaching the target (see methods). From 263 such recordings with active cells, there were 115 in which more than one simultaneously firing cell was isolated. The mean number of cells per recording level was 1.6 (range 1–4) and was slightly higher in the primary group (1.7) compared with the secondary static (1.5) or PANK2-NBIA groups (1.4), but this trend did not reach statistical significance. There was no significant difference in the GPi or GPe cell firing frequency between those patients who were on antidystonia medications at the time of surgery and those who were not (Mann Whitney U test p=0.401 and p=0.573, respectively).

**Table 1:** Number and firing frequency of cells by subgroup

	Primary		Secondary static		Secondary NBIA	
Participants, (n)	14		22		8	
Median age in years (range)	13.4 (4.5–18.1)		10.5 (3.3–17.8)		10.1 (4.3–17.2)	

	GPi cells	GPe cells	GPi cells	GPe cells	GPi cells	GPe cells

Cells, (n)	123	31	109	39	31	17
Median firing frequency (Hz)	13.5	13.5	9.6	7	25	15.9
Minimum–maximum frequency (Hz)	1–112	2–115	1–66	1–75	6–82	4–72
IQR	8.0–29.5	5.1–24.3	3.2–23.5	6.0–24.4	12.9–38.3	3.3–19.4

GPe, globus pallidus externa; GPi, globus pallidus interna; NBIA, neuronal brain iron accumulation.

### GPi cells

The distribution of GPi cell firing frequencies is shown in figure 2A (whole cohort) and figure 2B (subgroup). The difference in the GPi cell firing frequency across the groups was highly significant (figure 2C p=0.00003). The median GPi firing frequency was significantly higher for the primary group than for the secondary static group (p=0.002) and significantly higher for the NBIA group than for either the primary group (p=0.006) or the secondary static group (p=0.00004).

**Figure 3:**
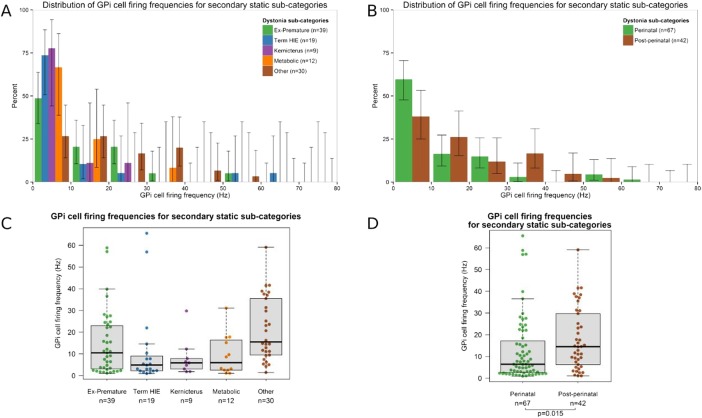
Distribution of GPi cell firing frequencies for the secondary static dystonia subgroups. (A and B) Histograms show the percentage of GPi cells within each 10 Hz frequency bin for each of the five aetiological subgroups (A) as well as for the perinatal onset and postperinatal onset groups (B). Lines show the 95% Agresti-Coull add-4 CIs. (C and D) Box-and-whisker plot of GPi cell firing frequencies for each of the five aetiological subgroups (C) as well as for the perinatal onset and postperinatal onset groups (D). Circles show the firing frequency of individual cells. Horizontal lines within boxes show the group median, boxes show the IQR, and whiskers show the full range. GPi, globus pallidus interna.

The secondary static group was further subdivided to compare the distribution of GPi cell firing frequencies by aetiology (figure 3A, C). In view of the small size of these subgroups, the patients were reclassified based on the timing of the lesion, either as perinatal onset (ex-premature, term HIE or kernicterus) or postperinatal onset (figure 3B, D). The GPi firing rate was significantly lower in the perinatal onset group compared with the postperinatal onset group (medians 6.5 Hz vs 14.6 Hz, p=0.022).

### GPe cells

The distribution of GPe cell firing frequencies is shown in figure 2D (whole cohort) and figure 2E (subgroup). The difference in the GPe cell firing frequency across the groups was significant (figure 2F, p=0.030). The median GPe firing frequency was significantly higher for the NBIA group than for the secondary static group (p=0.013) and significantly higher for the primary group than for the secondary static group (p=0.027).

### Patterns of cell firing

**Figure 4:**
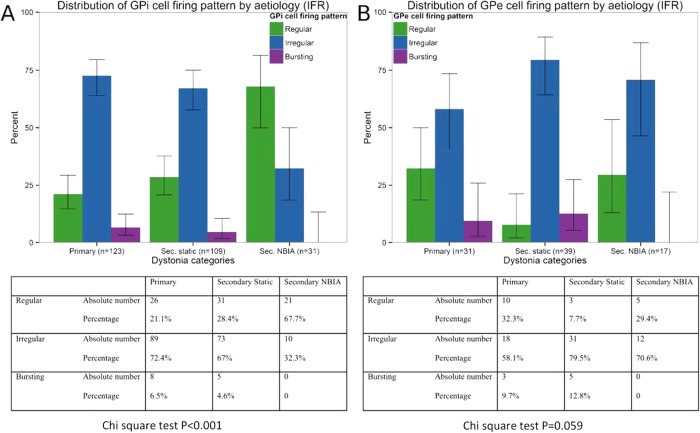
Pattern of GPi and GPe cell firing. Bar charts showing percentages of (A) GPi cells and (B) GPe cells for each dystonia group that were classified as regular, irregular or bursting. Tables show absolute numbers and percentages of cells in each category. χ^2^ Tests showed a significant difference in the pattern of cell firing across the three main groups for the GPi cells but not for the GPe cells. GPe, globus pallidus externa; GPi, globus pallidus interna.

The 263 GPi cells were classified as regular (n=78; 29.3%), irregular (n=172; 65.9%) or bursting (n=13; 4.8%). For the primary and secondary static groups, most cells were non-regular, whereas for the NBIA group most cells were regular (figure 4A). This difference in the GPi firing pattern was statistically significant (p=0.00006). The 87 GPe cells were classified as regular (n=18; 20%), irregular (n=61; 71.1%) and bursting (n=8; 8.9%). There was no significant difference in the GPe firing pattern across the three subgroups (figure 4B; p=0.059). Analysis of regularity using the ISI-CV method showed similar results (see online supplementary figure S2).

### Firing rates and patterns in relation to clinical phenotype

In addition to analysis by aetiology, pallidal firing rates and patterns were also compared across clinical phenotypes. For each patient, the clinical phenotype was classified as predominantly fixed/tonic dystonia or phasic/dynamic dystonia. Of the 41 patients with active cells, 24 had fixed/tonic dystonia and 17 had phasic/dynamic dystonia. The median GPi firing rate was 14.6 Hz (n=163 cells) for patients with predominantly fixed/tonic dystonia and 11.1 Hz (n=100 cells) for patients with phasic/dynamic dystonia. The Mann Whitney test showed that this small difference was significant (p=0.029). However, there is potential confounding here with aetiology since all patients with NBIA had fixed dystonia and it is clear that pallidal firing in these patients behaves differently from the other groups (see figures 2, 4 and online supplementary figure S1 and S2). When these patients are excluded from the cohort, the median GPi firing rate showed no significant difference between the Fixed (12.7 Hz) and phasic groups (11.1 Hz).

For the firing pattern, irregular and bursting cells were evenly distributed across the fixed and phasic groups, but regular cells were seen most commonly in the fixed group (χ^2^ p<0.001). Again, there is potential confounding here since a higher proportion of regular cells was seen in the NBIA group. However, when these patients were excluded, there was still a significantly higher proportion of regular cells in the Fixed as opposed to the Phasic dystonic group (χ^2^ p=0.030). No significant differences were observed in the GPe firing rate or pattern between the fixed and phasic groups.

### GPi and GPe firing rates in relation to severity and outcome

The relationship between severity (baseline BFMDRS-m score) and the GPi or GPe mean firing frequency within each individual was assessed using Spearman correlations. Patients in whom zero GPi or GPe cells were identified were excluded from the GPi or GPe correlation analysis, respectively. Lower GPi firing frequencies were associated with greater severity, but this trend did not reach statistical significance (n=39; Spearman's r −0.247; p=0.129). On subgroup analysis, a similar trend towards a negative correlation was observed for the secondary static group (n=21; r −0.406; p=0.068) but not for the primary (n=11; r 0.227, p=0.502) or secondary NBIA group (n=7; r 0.071, p=0.879). A significant negative correlation was seen between the mean GPi frequency and PLD for the group overall (n=39; r −0.362; p=0.023), but not for any of the subgroups.

**Figure 5:**
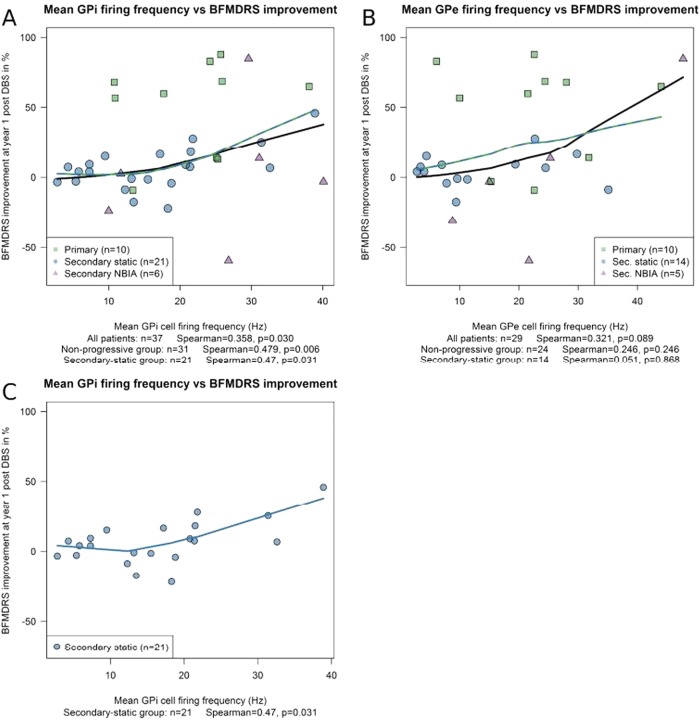
Relationship between the GPi and GPe firing frequency and DBS outcome. Scatterplots show the outcome 1 year after DBS, measured as the percentage improvement in BFMDRS-m score, against (A) the mean GPi firing frequency and (B) the mean GPe firing frequency for each patient. Individual patients from each subgroup are shown as per key. LOESS (non-parametric regression) curves illustrate the relationship for the group overall (black lines) and for the non-progressive group (blue and green lines). Note that while the curve in subfigure (B) appears to show a positive relationship, this relationship is only supported by a few observations and is not significant. (C) Shows outcome 1 year after DBS against the mean GPi firing frequency for the secondary static subgroup alone. BFMDRS-m, Burke-Fahn-Marsden Dystonia Rating Scale; DBS, deep brain stimulation; GPe; globus pallidus externa; GPi, globus pallidus interna.

One-year outcome data (% improvement in the BFMDRS-m score) were available for all except two patients, who had leads removed due to infection (see online supplementary table S1). A significant positive correlation was found between DBS outcome and GPi firing frequency (n=37; r 0.358, p=0.030) (figure 5A). Since children with NBIA have a degenerative condition and have clearly different pallidal firing characteristics from the other groups (figures 2, 4 and online supplementary figures S1 and S2), the analysis was repeated with this group excluded, resulting in an even stronger correlation between outcome and GPi firing frequency (n=31; r 0.479, p=0.006). A further consideration is that the relationship between GPi frequency and outcome could be confounded by the known differential response to DBS according to aetiology (generally higher levels of improvement being seen in patients with primary than secondary dystonia) and the finding that patients with secondary static dystonia show significantly lower GPi firing rates than those with primary dystonia (figure 2). The correlation analysis was therefore performed on each subgroup separately. This revealed a significant correlation between the mean GPi firing rate and outcome for the secondary static group (n=21; r 0.470, p=0.031) (figure 5C) but not for the primary (n=10; r 0.297, p=0.405) or NBIA group (n=6; r 0.371, p=0.468). A subgroup of patients with normal basal ganglia on MRI (n=18) also showed a trend towards a positive correlation between the mean GPi firing rate and outcome (n=18; r 0.432, p=0.073). Comparable results for these analyses were obtained when using the absolute change (rather than % change) in the BFMDRS-m score.

No statistically significant correlations were found between the GPe firing frequency and outcome for either the whole cohort (n=29; r 0.321, p=0.089, figure 5B) or the subgroups.

Finally, we found a positive correlation between GPi and GPe firing frequencies across the cohort as a whole (n=29 Spearman's r 0.439, p=0.017). On subgroup analysis, the direction of this relationship was maintained for each group, but the correlations were not significant.

## Discussion

We report the largest cohort of pallidal neuronal recordings in children (age 3–18) and in patients with secondary dystonia of any age, and the first cohort of pallidal recordings in NBIA. Our data reveal several new and important findings:

1. The GPi firing rate varies significantly with dystonia aetiology: the secondary static group overall showed significantly lower frequencies than the primary group (figure 2), whereas the NBIA group showed significantly higher GPi firing rates than either the primary or secondary Static group. Subdivision of the secondary static patients by aetiology revealed further differences: perinatal-onset patients, particularly the Term HIE and kernicterus groups, had significantly lower GPi firing rates than those with later onset (figure 3).
2. The GPe firing rate also differs with dystonia aetiology: the NBIA group showed significantly higher GPe firing rates than the Secondary Static group, while the primary group showed a trend towards higher GPe firing rates than the secondary static group (figure 2F).
3. The GPi firing *pattern* differs with dystonia aetiology. Our primary and secondary static groups showed predominantly irregular or bursting cells (figure 4A), whereas our NBIA group had predominantly *regular* GPi cells.
4. Regular GPi firing patterns correlated with fixed/tonic dystonia phenotypes, that is, a sustained GPi firing pattern is consistent with a sustained, rather than fluctuating, dystonia.
5. The GPi firing rate shows a positive correlation with DBS outcome at 1 year (figure 5A), which was most striking for the patients with non-progressive dystonia, particularly the secondary static group (figure 5C).

### Comparison with previous studies

The number of cells recorded per trajectory is similar to that in previous studies.^22^ The finding of fewer GPe than GPi cells in most patients with primary and secondary static dystonia also accords with adult findings.^22^ ^26^ Overall, the pallidal firing rates observed in this study (mean of 19.1 Hz) are lower than those reported previously (means of 23.4–55.3 Hz).^23–27^ This may, in part, reflect the light isoflurane anaesthesia,^33^ ^34^ but other factors may have contributed; for example (1) some studies excluded recordings with less than 800 spikes,^22^ which will automatically exclude lower firing cells, increasing the mean firing rate; (2) we had a larger number of patients with secondary dystonia who tend to have lower firing rates; (3) our cohort included more severely affected patients and more patients with perinatal brain injury than most previous studies. There is some evidence that GPi firing correlates inversely with severity^22^ (although our own data suggest that this does not apply to the NBIA group), and our own data demonstrate that the lowest firing rates were seen in patients with dystonia secondary to perinatal brain injury (figure 3). These factors are difficult to tease apart since the patients with perinatal brain injury are often among the most severe, but either or both of these factors would tend to reduce the overall firing rates in our cohort.

However, the reduced firing rate of GPi neurons in dystonia in general is not the focus here. This has been demonstrated in many other papers from data collected without an anaesthetic.^23–27^ The focus of *our* report is comparisons between different types of dystonia. The fact that all patients received the same anaesthetic regimen means that differences observed between patients cannot be attributed solely to the effect of the anaesthetic. Although the possibility that patients with different conditions may respond differently to anaesthetic agents is a consideration, this is unlikely since other reports indicate that general anaesthesia reduces pallidal firing rates in dystonia of different aetiology^33^ and distribution^35^ and in Parkinson's disease.^26^ Therefore, the intergroup differences observed in our study are likely to reflect genuine pathophysiological differences. This is supported by several observations: (A) we found a trend towards a negative correlation between the GPi firing rate and severity of dystonia. A similar relationship has been demonstrated in data collected without an anaesthetic.^22^ (B) We found lower GPi firing rates in secondary compared with primary dystonia. Again, a similar relationship has been demonstrated in data collected without an anaesthetic,^22^ albeit with only three patients with secondary dystonia. (C) We found a significant positive correlation between the GPi firing rate and outcome from DBS. Thus, although anaesthesia may have reduced the GPi firing rate across the cohort as a whole, the relative differences in firing rates between patients must have been preserved; otherwise, this relationship could not have been identified.

### Relevance

The underlying mechanisms of dystonia are still incompletely understood. The basal ganglia are clearly implicated in the pathophysiology but the actual mechanisms are unknown,^36^ and there is growing evidence for involvement of other brain regions including the cerebellum and sensorimotor cortex.^37^ Reduced inhibition has been demonstrated at many levels of the nervous system in primary dystonia.^38–40^ The finding of reduced GPi firing rates in dystonia^22–27^ is consistent with reduced inhibitory output from the basal ganglia, in turn resulting in excess unwanted motor activity. There is evidence that synchronised low frequency pallidal activity plays a role in primary dystonia.^41–43^ DBS suppresses this activity in patients with a predominantly phasic dystonia.^43^ However, it is still unclear how DBS improves motor symptoms, and why there is such a wide variation in patient benefit between primary and secondary dystonia. This differential response to DBS points towards differences in the underlying mechanisms between primary and secondary dystonia.^36^ The clear differences in pallidal firing rates and patterns that we demonstrate between different dystonia aetiologies add to our understanding of these different pathophysiological mechanisms. The spontaneous firing of pallidal neurones may well reflect intrinsic neuronal properties that influence the responsiveness to pallidal stimulation.^44^

Previous studies found no difference in GPi firing rates between primary and secondary dystonia,^26^ ^27^ except one study which included only three patients with secondary dystonia.^22^ Our secondary group (n=31) was much larger than that in previous studies (n=3–9), providing us adequate power to detect a difference. We also subdivided patients by aetiology, revealing different firing rates between dystonia groups (figure 2). This electrophysiological distinction between subtypes of secondary dystonia may explain the absence of differences between primary and secondary dystonia in the small numbers of cases reported previously.^26^ ^27^ Our secondary static group was heterogeneous but subdivision indicated further differences between aetiological groups which are likely to become more apparent with larger studies (figure 3). We also demonstrated a link between regular firing patterns and a fixed/tonic dystonia phenotype. This has potential clinical importance since this group tends to show smaller improvements with DBS than patients with predominantly phasic movements.^17^ ^43^

Predicting which patients will benefit from DBS is particularly difficult among patients with secondary dystonia, in whom the outcome varies widely.^17^ Our finding of a correlation between the mean GPi firing rate per individual and DBS outcome is therefore highly relevant. Lower mean spontaneous GPi firing rates were associated with less improvement in the non-progressive patients (figure 5A). This was not simply a reflection of the lower firing rates seen in the secondary static subgroup compared with patients with primary dystonia, since a significant correlation was observed even *within* the secondary static subgroup (figure 5C). This relationship, although needing confirmation from larger studies, has potential clinical application in the management of patients with secondary dystonia. Where intraoperative recordings predict a worse outcome, this could allow the search, within the same operative session, for possible alternative targets with higher neuronal firing frequency that might confer a greater likelihood of benefit from DBS. This could include determining an ‘optimal position’ within the GPi based on a richer firing pattern, or opting for subthalamic nucleus (STN) targeting if the GPi firing pattern is found to be of very low frequency. This would, of course, need to be considered at the preoperative planning stage, so that the alternative target could be approached within a single surgery.

Our study includes the largest reported cohort of PANK2-NBIA children with pallidal microelectrode recordings. (Microelectrode data from only one NBIA patient have been reported previously).^22^ ^45^ The finding of higher GPi firing rates in NBIA is in keeping with resting state positron emission tomography with 2-deoxy-2-[fluorine-18]fluoro-D-glucose integrated with CT (FDG-PET-CT) brain scan data (obtained during uptake) showing high FDG-glucose metabolic activity in the posterior putamen in NBIA compared with primary dystonia.^46^ NBIA is often considered a mixed motor disorder comprising features of Parkinsonism and dystonia, so the high, regular pallidal firing we observed in NBIA is interesting in view of the higher GPi firing rates documented in adult Parkinson's disease.^22^ This is consistent with the relative akinesia seen in our children with PANK2-NBIA.

The presence of spontaneous irregular and bursting GPi activity is a feature of both dystonia and Parkinson's disease in adults,^22^ so our observation of a reversed pattern with more regular than irregular cells in PANK2-NBIA is intriguing. We also found a lower ratio of GPi to GPe cells in the NBIA group (see online supplementary materials). We speculate that this might reflect selective loss of a subpopulation of GPi cells, leaving a subpopulation of faster firing, regular GPi cells lacking variability, thus altering the behaviour of indirect pathway physiology in favour of akinesia. Pralong *et al*^47^ report two cases of Lesch-Nyhan disease in which higher firing rates were identified in the GPe than GPi, leading to the hypothesis that inhibition of GPi output by dominant GPe firing might cause dystonia. We did not observe such a relationship in our data set. Although we observed some individuals in whom the mean GPe firing rate exceeded the mean GPi firing rate, we observed more individuals with the opposite relationship and, overall, we found a positive rather than negative correlation between GPi and GPe firing frequencies across the cohort. On subgroup analysis, the direction of this relationship was maintained for each group, but the correlations were not significant (possibly related to smaller numbers). This difference in findings may in part reflect methodological factors but could also support the concept of disease-specific pallidal firing patterns, as there were no Lesch-Nyhan cases within our cohort.

Interestingly, patients with PANK2-NBIA, despite their relatively higher GPi firing rates, do not show a sustained superior therapeutic response to DBS compared with primary dystonia.^7^ ^48^ ^49^ Patients with PANK2-NBIA often do respond well initially but then deteriorate again, probably reflecting the progressive nature of their condition. Our experience has been 6 months to 2 years of good responsiveness to DBS followed by a gradual return of often quite severe symptoms, particularly in the early onset cases. Our findings indicate that pallidal neurones in PANK2-NBIA behave quite differently from those in patients with dystonia of various other aetiologies, indicating a different pathophysiology of PKAN versus other dystonias. Such disease-specific firing patterns could be an important biomarker for future closed loop systems, as are currently being investigated in Parkinson's disease^50^ or for disease-modifying strategies such as gene-vector therapies. Furthermore, the striking differences in pallidal firing for PANK2-NBIA compared with other dystonia patients, alongside the FDG-PET-CT imaging patterns, could warrant the exploration of alternative or additional neuromodulation targets in this group, such as the subthalamic nucleus.

### Limitations

1. The study was retrospective. However, all analyses of neuronal recordings and positions within the GPe/GPi were performed blind to the patients’ clinical details. Clinical assessments were blind to the microelectrode data.
2. Data were recorded under an anaesthetic. However, intergroup differences are unlikely to be related to this (see above). Differences in pallidal activity between patients with primary dystonia and patients with DYT11 myoclonus-dystonia have recently been demonstrated by other authors in patients under anaesthesia.^51^
3. Single-pass microelectrode recordings were performed (to reduce potential haemorrhage risk), yielding fewer trajectories of data than some studies. This limited our ability to perform more detailed mapping or to compare findings from several trajectories within a given subject.
4. We did not perform local field potentials, which would provide valuable additional information. However, the relationship of our data to outcome clearly demonstrates the importance of also analysing individual neuronal activities.

## Conclusions

We demonstrate that GPi and GPe firing rates and patterns correlate with dystonia aetiology and phenotype, providing further evidence of differences in the pathophysiology of primary versus secondary dystonia, including PANK2-NBIA. Clear delineation of these different mechanisms is essential for us to explain and address the relatively poorer response of patients with secondary dystonia to DBS. The finding of a positive correlation between the GPi firing rate and DBS outcome warrants exploration with further studies and has potential clinical implications for individualised protocols for neuromodulation in dystonia. The present findings make a significant contribution to our understanding of the disease-specific pathophysiology in dystonia. Ongoing work to further advance this knowledge is essential to facilitate the effectiveness of new technologies, such as closed-loop neuromodulation systems, in producing benefit for individual patients.

## Supplementary Material

Web supplement

Web table
