# Supplementary material for: Differences in globus pallidus neuronal firing rates and patterns relate to different disease biology in children with dystonia
Source: J Neurol Neurosurg Psychiatry. 2016 Feb 4;87(9):958–67. doi: 10.1136/jnnp-2015-311803 (PMC5013118; doi:10.1136/jnnp-2015-311803)
Supplement: Web supplement [file jnnp-2015-311803-s1.pdf]

Appendix of Supplementary material

Differences in globus pallidus neuronal firing rates relate to dystonia aetiology in children

VM McClelland, A Valentin, HG Rey, DE Lumsden, MC Elze, R Selway, G Alarcon, JP Lin

Table of Contents

Statistical methods for supplementary multilevel modelling 1
Balance of GPi and GPe cells…………………………………………………………………………..3
Pattern of GPi and GPe cell firing assessed by ISI-CV method………………………………………………...4

Supplementary tables

Multilevel modelling of GPi cell firing frequency across dystonia sub-groups 1
Multi-level modelling analysis of GPe cell firing frequency across dystonia sub-groups 2
Multilevel modelling of the proportion of GPi and GPe cells across dystonia sub-groups............................... 2

Supplementary figures

Figure S1: GPi/GPe cell ratio……………………………………………………………………………..….3
Figure S2: Pattern of GPi and GPe cell firing assessed by ISI-CV method…………………………..…..…….4

Strategy for supplementary multilevel modelling

GPi and GPe cells belong to individual patients. Cells belonging to the same patient cannot be assumed to be fully independent as e.g. a patient might have a higher than usual proportion of GPi cells. In addition, there may be effects due to the measurement procedure such as the trajectory chosen. To investigate such effects, multilevel modelling, also known as mixed models, is an appropriate tool. We reanalyse the data from Figures 2 and S1 to verify that the conclusions hold up.

For Fig. 2, linear mixed models were fitted. For Fig. S1, mixed models with a logistic link function were fitted for the probability of observing a GPi instead of a GPe cell. All models included patient-level random effects. Except when comparing nested models, a restricted maximum likelihood (REML) approach was used. To assess which variables should be included in the final model, p-values were calculated using 19999 parametric bootstrap runs. Variable selection using step-up and step-down procedures resulted in the same final models.

Computations were performed using the R language and environment for statistical computing (version 3.01) together with the lme4 package for linear mixed-effects models (version 1.1-6), the pbkrtest package for the (version 0.3-7), and the afex package for the analysis of factorial experiments (version 0.12-135).

Multilevel modelling of GPi cell firing frequency across dystonia sub-groups

For GPi cells, the dystonia aetiology was found to be a significant predictor of mean firing frequency (p=0.00385). Brain side (p=0.27448), electrode position (p=0.52275) and firing pattern (p=0.38124) were not found to be significantly associated with mean firing frequency and were excluded from the final model. The results match our findings in Fig. 2.

	**Estimate**	**SE**
**(Intercept)**	2.5998	0.1404
**Sec. static**	-0.4100	0.1892
**Sec. NBIA**	0.5221	0.2668

Table S2. Fixed effect estimates of the final multilevel model for log(GPi cell mean firing frequency). Patients with primary dystonias are the baseline in this model. All values rounded to 4 digits.

Groups	Variance	SD
**Patient ID**	0.1132	0.3365
**Residual**	0.9198	0.9591

Table S3. Variance and standard deviation for random effect estimates of the final multilevel model for log(GPi cell mean firing frequency). Number of observations: 263. Number of patients: 39. All values rounded to 4 digits.

Multi-level modelling analysis of GPe cell firing frequency across dystonia sub-groups

For GPe cells, the dystonia aetiology was found to be a significant predictor of mean firing frequency (p=0.01128). Brain side (p=0.26733), electrode position (p=0.62691) and firing pattern (p=0.48000) were not found to be significantly associated with mean firing frequency and were excluded from the final model. The results match our findings in Fig. 2.

	Estimate	SE
**(Intercept)**	2.5745	0.1867
**Sec. static**	-0.6204	0.2504
**Sec. NBIA**	0.2402	0.3137

Table S4. Fixed effect estimates of the final multilevel model for log(GPe cell mean firing frequency). Patients with primary dystonias are the baseline in this model. All values rounded to 4 digits.

Groups	Variance	SD
**Patient ID**	0.0040	0.0632
**Residual**	1.0649	1.0319

Table S5. Variance and standard deviation for random effect estimates of the final multilevel model for log(GPe cell mean firing frequency). Number of observations: 87. Number of patients: 30. All values rounded to 4 digits.

Multilevel modelling of the proportion of GPi and GPe cells across dystonia sub-groups

When comparing the probability of observing a GPi cell (as opposed to a GPe) cell, aetiology was found to be a significant predictor (p=0.01620). The results match our findings in Fig. S1.

	Estimate	SE
**(Intercept)**	1.3802	0.2192
**Sec. static**	-0.4255	0.2981
**Sec. NBIA**	-1.2004	0.3600

Table S6. Fixed effect estimates of the final binomial multilevel model for probability of observing a GPi cell. Patients with primary dystonias are the baseline in this model. All values rounded to 4 digits.

Groups	Variance	SD
**Patient ID**	0.06122	0.2474

Table S7. Variance and standard deviation for random effect estimates of the final binomial multilevel model for probability of observing a GPi cell. Number of observations: 41. Number of patients: 41. All values rounded to 4 digits. Residual variance is not available with a logit link function.

Balance of GPi and GPe cells

The balance between the number of GPi and GPe cells across the groups was assessed by calculating the ratio between the number of GPi cells per trajectory and the number of GPe cells per trajectory for each individual (the “GPi-GPe cell ratio”). (Patients in whom zero GPi or GPe cells were identified were excluded from this analysis since the ratio could not be calculated). The median GPi-GPe cell ratios were 4.0 for the Primary (n=10), 2.5 for Secondary Static (n=14) and 1.0 for the NBIA groups (n=5) (Figure S1). The difference across groups was significant (Kruskal-Wallis test p=0.009) with a smaller ratio seen for the NBIA group than the Primary group (Mann-Whitney test p=0.002).

No significant difference was seen in the GPi versus GPe cell firing frequency for any group although for the NBIA group there was a trend towards a higher GPi than GPe firing frequency (medians 25Hz vs 15.9Hz respectively) (Mann-Whitney test p=0.074).

The relative balance of GPi to GPe cells also varies with dystonia aetiology (Fig. S1). In most primary and secondary static patients the recorded GPi cells outnumbered the GPe cells, similar to adult findings, [19 23] whereas our NBIA group showed a more balanced ratio of GPi and GPe cells (Fig. S1) and a trend towards a higher GPi than GPe firing frequency.

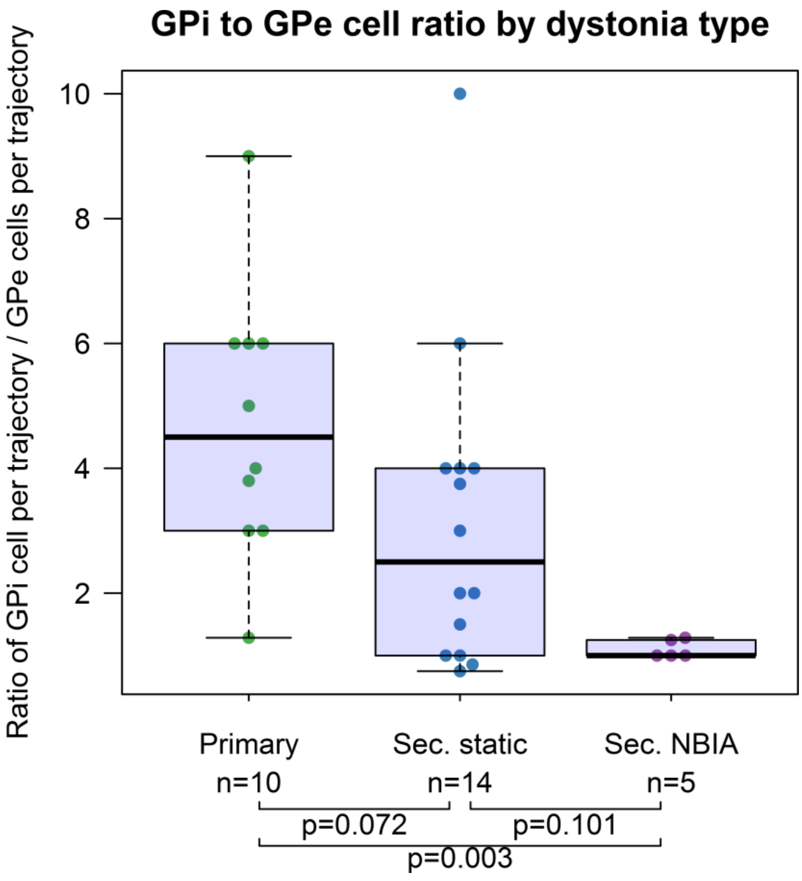

Fig. S1. Ratio of GPi to GPe cells

Box-and-whisker plot of the ratio of the number of GPi cells per trajectory to the number of GPe cells per trajectory for patients in each of the three main dystonia groups. Circles show ratios for individual patients. Horizontal lines within boxes show the group median, boxes show interquartile range, whiskers show full range excluding outliers. Kruskal-Wallis test showed a significant difference in median GPi-GPe cell ratio across the 3 groups (p=0.009). The p-values show the results of Mann-Whitney tests between groups.

Pattern of GPi and GPe cell firing assessed by ISI-CV method

To investigate whether our results in Fig. 4 depend on the choice of the IFR versus the ISI-CV method for assessment, we repeated the analysis in Fig. 4 using the ISI-CV method for assessment. While the results differ to some extent, the key conclusions in Fig. 4 are unaffected by the choice of method.

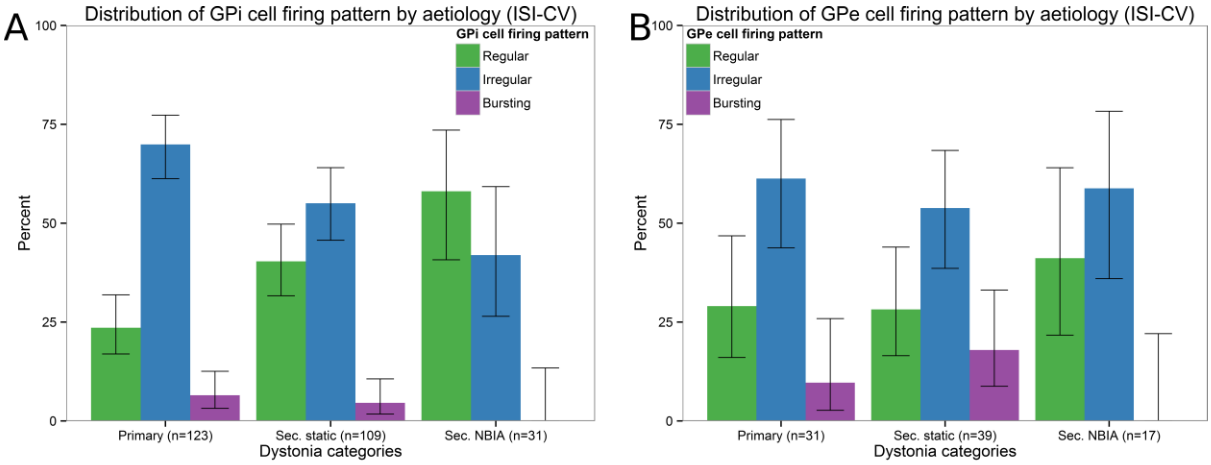

		Primary	Secondary Static	Secondary NBIA
Regular	Absolute number	29	43	18
	Percentage	23.6%	39.4%	58.1%
Irregular	Absolute number	86	61	13
	Percentage	69.9%	56%	41.9%
Bursting	Absolute number	8	5	0
	Percentage	6.5%	4.6%	0

Chi square test P=0.003

		Primary	Secondary Static	Secondary NBIA
Regular	Absolute number	9	11	7
	Percentage	29%	28.2%	41.2%
Irregular	Absolute number	19	21	10
	Percentage	61.3%	53.8%	58.8%
Bursting	Absolute number	3	7	0
	Percentage	9.7%	17.9%	0

Chi square test P=0.488

Figure S2. Pattern of GPi and GPe cell firing assessed using the ISI-CV method
Bar charts showing percentages of (A) GPi cells and (B) GPe cells for each dystonia group that were classified as regular irregular or bursting. Tables show absolute numbers and percentages of cells in each category. Chi-square tests showed a significant difference in the pattern of cell firing across the three main groups for the GPi cells but not for the GPe cells.
