# Supplementary material for: Differences in globus pallidus neuronal firing rates and patterns relate to different disease biology in children with dystonia
Source: J Neurol Neurosurg Psychiatry. 2016 Feb 4;87(9):958–67. doi: 10.1136/jnnp-2015-311803 (PMC5013118; doi:10.1136/jnnp-2015-311803)
Supplement: Web table [file jnnp-2015-311803-s2.pdf]

| Patient | Age at DBS | Male (M) or Female (F) | Dystonia Group | Age at onset (years) | Distribution of dystonia | Phenotype: Fixed or Phasic | Inherited, Acquired, Idiopathic | Aetiology- | Imaging | Medication at time of surgery | Duration of Dystonia (years) | Proportion of Life Lived with dystonia | Baseline BFMDRS motor score | 1 year BFMDRS motor score | % Improvement at 1 year |
|---|---|---|---|---|---|---|---|---|---|---|---|---|---|---|---|
| 1 | 13.9 | M | Primary | 10.9 | Generalised. Neck, trunk and all 4 limbs | Phasic | Inherited | DYT1 positive | Normal | None (previous either ineffective or side effects) | 3 | 0.22 | 57 | 23 | 59.6 |
| 2 | 11.8 | M | Primary | 8 | Generalised, particularly both legs and left arm | Fixed | Inherited | DYT1 positive | Normal | Co-Careldopa | 3.8 | 0.32 | 59.5 | 10 | 83.2 |
| 3 | 7.3 | F | Primary | 6.3 | Generalised, particularly both legs and right arm | Fixed | Inherited | DYT1 positive | Normal | Trihexyphenidyl | 1 | 0.14 | 50 | 6 | 88.0 |
| 4 | 10.8 | M | Primary | 3.5 | Generalised | Fixed | Idiopathic | Idiopathic (DYT1, DYT5 and PANK2 negative) | Normal | None | 7.3 | 0.68 | 75 | 26.5 | 64.7 |
| 5 | 13.9 | M | Primary | 11.5 | Generalised | Fixed | Inherited (positive family history) | Idiopathic | Normal | Diazepam | 2.4 | 0.17 | 48 | 15.5 | 67.7 |
| 6 | 12.9 | M | Primary (plus) | 2.9 | Generalised – particularly face and upper limbs | Phasic | Inherited | DYT11 | Normal | None | 10 | 0.78 | 30 | 9.5 | 68.3 |
| 7 | 17.1 | F | Primary (plus) | 14.1 | Predominantly right upper limb | Phasic | Inherited | Idiopathic (DYT11 and TITF1 negative) | Normal | None | 3 | 0.18 | 8 | 1 | 87.5 |
| 8 | 12.2 | F | Primary (plus) | 0.2 | Generalised | Phasic | Idiopathic | Idiopathic | Normal | None | 12 | 0.98 | 34 | 35 | -2.9 |
| 9 | 8.9 | M | Primary (plus) | 0.5 | Generalised | Fixed | Idiopathic | Unknown | Normal | Baclofen<br>Trihexyphenidyl | 8.4 | 0.94 | 64.5 | 55.5 | 14.0 |
| 10 | 15.9 | F | Primary (plus) | 0.6 | Generalised | Phasic | Idiopathic | Unknown | Normal | None | 15.3 | 0.96 | 59 | N/A * | N/A * |
| 11 | 13.1 | F | Primary (plus) | 9.1 | Generalised | Phasic | Idiopathic | Unknown | Normal | Intrathecal baclofen<br>Trihexiphendidyl<br>Diazepam | 4 | 0.31 | 89.5 | 78 | 12.8 |
| 12 | 17.4 | F | Primary (plus) | 4.4 | Generalised but predominantly upper limbs (R>L) and R lower limb. | Phasic | Idiopathic | Unknown | Normal basal ganglia.<br>Non-specific left Hippocampus abnormality | None | 13 | 0.75 | 38 | 16.5 | 56.6 |
| 13 | 4.5 | M | Primary (plus) | 0.8 | Generalised | Phasic | Idiopathic | Unknown | Normal basal ganglia.<br>Non-specific left frontal arachnoid cyst | Lorazepam<br>Tizanidine<br>Trihexyphenidyl | 3.7 | 0.82 | 88.5 | 96.5 | -9.0 |
| 14 | 18.1 | M | Primary (plus) | 9 | Generalised | Fixed | Idiopathic | Unknown | Normal | Sinemet plus | 9.1 | 0.50 | 24 | 31 | -29.2 |
| 15 | 12.6 | F | Secondary static | 0.6 | Generalised | Fixed | Acquired | Ex-premature 26/40 (Perinatal brain injury) | Thalamus, PVWM | Baclofen<br>Nitrazepam | 12 | 0.95 | 103 | 86 | 16.5 |
| 16 | 7.9 | M | Secondary static | 0.9 | Generalised | Fixed | Acquired | Ex premature 27/40 (Perinatal brain injury) | PVWM | Baclofen<br>Clonidine<br>Trihexiphenidyl Dantrolene | 7 | 0.89 | 106 | 109.5 | -3.3 |
| 17 | 6.1 | F | Secondary static | 0.1 | Generalised | Phasic | Acquired | Ex-premature 24/40 (Perinatal brain injury) | PVWM | Trihexiphenidyl | 6 | 0.98 | 106.5 | 97 | 8.9 |
| 18 | 10.6 | M | Secondary static | 0 | Generalised | Fixed | Acquired | Ex-premature 25 /40 (Perinatal brain injury) | Normal | Trihexiphenidyl | 10.6 | 1.00 | 87.5 | 84 | 4.0 |
| 19 | 9.2 | M | Secondary static | 0.2 | Generalised | Phasic | Acquired | Ex-premature 32/40 twin, twin-twin transfusion donor, neonatal sepsis (Perinatal brain injury) | Signal change and volume loss in Globi pallidi and PVWM | Clonazepam | 9 | 0.98 | 109.5 | 101.5 | 7.3 |
| 20 | 5.5 | F | Secondary static | 0 | Generalised | Phasic | Acquired | Ex premature 25/40 twin (Perinatal brain injury) | White matter volume loss + Focal right cerebellar cortical dysplasia | None | 5.5 | 1.00 | 110 | 111 | -0.9 |
| 21 | 12.1 | M | Secondary static | 0.1 | Generalised | Fixed | Acquired | (Perinatal brain injury) Ex-premature 28/40 G6PD deficiency leading to hyperbilirubinaemia | Basal gangla normal<br>Minimal white matter volume loss, | Baclofen<br>Trihexiphenidyl Nitrazepam, Haloperidol | 12 | 0.99 | 114.5 | 106 | 7.4 |
| 22 | 16.8 | F | Secondary static | 0.8 | Generalised | Phasic | Acquired | Term HIE (Perinatal brain injury) | Gliosis of thalami, lentiform nuclei and periorolandic cortex. | Trihexiphenidyl | 16 | 0.95 | 107 | 110 | -2.8 |

| | | | | | | | | | | | | | | | |
|---|---|---|---|---|---|---|---|---|---|---|---|---|---|---|---|
| 23 | 13.7 | M | Secondary static | 1.7 | Generalised | Fixed | Acquired | Term HIE<br>(Perinatal brain injury) | Gliosis of thalami, lentiform nuclei and periorolandic cortex | Trihexiphenidyl<br>Baclofen | 12 | 0.88 | 102.5 | 93 | 9.3 |
| 24 | 17.8 | F | Secondary static | 0.8 | Generalised | Phasic | Acquired | Term HIE<br>(Perinatal brain injury) | Gliosis of thalami, lentiform nuclei and periorolandic cortex | Baclofen | 17 | 0.96 | 72 | 73 | -1.4 |
| 25 | 7.2 | M | Secondary static | 0 | Generalised | Phasic | Acquired | Term kernictuerus<br>(Perinatal brain injury) | Gliotic change in Globi pallidi consistent with previous elevated bilirubin | None | 7.2 | 1.00 | 66 | 77.5 | -17.4 |
| 26 | 3.5 | M | Secondary static | 0 | Generalised | Fixed | Acquired | Term kernictuerus<br>(Crigler-Najjar)<br>(Perinatal brain injury) | Gliotic change in Globi pallidi consistent with previous elevated bilirubin | Trihemiphenidyl<br>Nitrazepam | 3.5 | 1.00 | 98 | 106.5 | -8.7 |
| 27 | 12.3 | M | Secondary static | 0 | Generalised | Fixed | Acquired | Term kernictuerus<br>(Perinatal brain injury) | Signal change in Globus pallidi | Baclofen,<br>Trihexiphenidyl<br>Co-careldopa | 12.3 | 0.98 | 111.5 | 107 | 4.0 |
| 28 | 10.6 | F | Secondary static | 0.6 | Generalised | Fixed | Inherited – inborn error of metabolism | Glutaric aciduria type 1<br>(Toxic/metabolic) | Gliotic change and volume loss in basal ganglia and white matter | Baclofen<br>Chloral hydrate<br>Diazepam<br>Clonazepam Tetrabenazine | 10 | 0.94 | 112.5 | 95.5 | 15.1 |
| 29 | 15.7 | M | Secondary static | 1.7 | Generalised | Fixed | Inherited – inborn error of metabolism | Glutaric aciduria type 1<br>(Toxic/metabolic) | Gliotic change and volume loss in basal ganglia and white matter | Diazepam<br>Baclofen<br>Trihexyphenidyl | 14 | 0.89 | 114.5 | 105.5 | 7.9 |
| 30 | 10.4 | F | Secondary static | 3 | Left hemidystonia | Phasic | Acquired | Focal infarct<br>(Vascular) | Infarct right caudate and pallidum | None | 7.4 | 0.71 | 30.5 | 16.5 | 45.9 |
| 31 | 3.3 | M | Secondary static | 1.3 | Generalised | Fixed | Inherited – Acquired | Bilateral nigrostriatal necrosis, Aicardi Goutieres type 6<br>ADAR1 positive | Bilateral nigrostriatal necrosis | Diazepam<br>Baclofen<br>Trihexiphenidyl | 2 | 0.61 | 102.5 | 74 | 27.8 |
| 32 | 6.7 | M | Secondary static | 1.7 | Generalised | Fixed | Inherited - Acquired | Bilateral nigro-striatal necrosis, Aicardi Goutieres type 6. ADAR1 positive | Bilateral nigrostriatal necrosis | Baclofen<br>Carbamazepine | 5 | 0.75 | 83 | 62 | 25.3 |
| 33 | 13.1 | F | Secondary static | 0.1 | Generalised | Phasic | Uncertain | Unknown. Bilateral perisylvian polymicrogyria with epilepsy and dystonia | Malformation: Perisylvian polymicrogyria and cerebellar atrophy | Tetrabenazine<br>Trihexiphenidyl | 13 | 0.99 | 104 | 97 | 6.7 |
| 34 | 15.7 | M | Secondary static | 3.7 | Generalised but predominantly right sided | Fixed | Probably Acquired | Unknown | Left putaminal reduced volume | | 12 | 0.76 | 80 | 65.5 | 18.1 |
| 35 | 7.1 | M | Secondary static | 0.1 | Generalised | Fixed | Probably inherited: affected sister | Unknown | PVWM | Tetrabenazine<br>Co-careldopa | 7 | 0.99 | 97 | 101 | -4.1 |
| 36 | 8.9 | F | Secondary static | 6 | Generalised | Phasic | Probably inherited | Unknown.<br>Congential neuromuscular disorder: LMNA mutation | Normal | Trihexiphenidyl | 2.9 | 0.33 | 52.5 | 64 | -21.9 |
| 37 | 17.2 | F | Secondary progressive | 8.2 | Generalised | Fixed | Inherited – inborn error of metabolism | NBIA- PANK2 deficiency | Iron deposition in GP and nigro-striatal tract. | None | 9 | 0.52 | 59.5 | 78 | -31.1 |
| 38 | 7.9 | F | Secondary progressive | 5.9 | Generalised | Fixed | Inherited – inborn error of metabolism | NBIA- PANK2 deficiency | Iron deposition in GP and nigro-striatal tract. | Baclofen<br>Diazepam<br>Trihexiphenidyl | 2 | 0.25 | 64 | 102 | -59.4 |
| 39 | 10.5 | M | Secondary progressive | 5.5 | Generalised | Fixed | Inherited – inborn error of metabolism | NBIA- PANK2 deficiency | Iron deposition in GP and nigro-striatal tract. | Baclofen<br>Diazepam | 5 | 0.48 | 98 | 95.5 | 2.6 |
| 40 | 4.3 | M | Secondary Progressive | 1.5 | Generalised | Fixed | Inherited – inborn error of metabolism | NBIA- PANK2 deficiency | Iron deposition in GP and nigro-striatal tract. | Trihexiphenidyl<br>Haloperidol<br>Clonidine | 2.8 | 0.65 | 97.5 | N/A ** | N/A** |
| 41 | 13.6 | F | Secondary progressive | 3.6 | Generalised | Fixed | Inherited – inborn error of metabolism | NBIA- PANK2 deficiency | Iron deposition in GP and nigro-striatal tract. | Intrathecal baclofen | 10 | 0.74 | 120 | 18 | 85.0 |
| 42 | 9.6 | F | Secondary progressive | 3.1 | Generalised | Fixed | Inherited – inborn error of metabolism | NBIA- PANK2 deficiency | Iron deposition in GP and nigro-striatal tract. | Baclofen<br>Trihexiphenidyl<br>Gabapentin | 6.5 | 0.68 | 82.5 | 85 | -3.0 |
| 43 | 16.4 | M | Secondary | 4.4 | Generalised | Fixed | Inherited – inborn | NBIA- PANK2 deficiency | Iron deposition in GP and nigro- | Diazepam | 12 | 0.73 | 114 | 98.5 | 13.6 |

| | | | | | | | | | | | | | | | |
|---|---|---|---|---|---|---|---|---|---|---|---|---|---|---|---|
| | | | progressive | | | | error of metabolism | | striatal tract. | Baclofen<br>Tetrabenazine<br>Clonazepam | | | | | |
| 44 | 6 | M | Secondary progressive | 2 | Generalised | Fixed | Inherited – inborn error of metabolism | NBIA- PANK2 deficiency | Iron deposition in GP and nigro-striatal tract. | Co-Careldopa,<br>Trihexiphenidyl<br>Sodium valproate | 4 | 0.67 | 86.5 | 107 | -23.7 |

**Table S1 – Clinical Data for individual patients** PVWM = Periventricular White Matter signal change, GP = Globi pallidi

* patient had DBS electrodes removed 3 months post procedure due to infection

** patient had DBS electrodes removed 1 month post procedure to due brain abscess associated with coincident acute hepatitis A infection which had provoked status dystonicus prior to DBS surgery and followed by nephrotic syndrome after DBS surgery.
